# Patterns of failure and clinical outcomes of definitive radiotherapy for cervical esophageal cancer

**DOI:** 10.18632/oncotarget.15665

**Published:** 2017-02-24

**Authors:** Lina Zhao, Yongchun Zhou, Yunfeng Mu, Guangjin Chai, Feng Xiao, Lina Tan, Steven H. Lin, Mei Shi

**Affiliations:** ^1^ Department of Radiation Oncology, Xijing Hospital, Fourth Military Medical University, Xi’an, 710032, China; ^2^ Department of Radiation Oncology, The University of Texas MD Anderson Cancer Center, Houston, Texas 77030, USA

**Keywords:** definitive radiotherapy, cervical esophageal cancer, failure pattern, prognostic factor, prophylactic irradiation

## Abstract

**Purpose:**

Because of the scarcity of cervical esophageal cancer (CEC), data for this disease entity is limited. We aim to evaluate the outcomes, prognostic factors and failure patterns of CEC treated by contemporary radiotherapy (RT).

**Methods:**

We retrospectively analyzed 86 CEC patients consecutively treated between 2007 and 2015 by definitive RT with or without concurrent chemotherapy. RT was mainly delivered with Intensity Modulated Irradiation Therapy (IMRT) or Volumetric-Modulated Arc Therapy (VMAT). Statistical analyses were performed on survival, prognostic factors and failure patterns.

**Results:**

The median follow-up time was 19.4 months. The 3-year overall survival (OS), local regional failure free survival (LRFFS), distant metastatic free survival (DMFS), and progression free survival (PFS) were 53.6%, 57.9%, 81.5% and 41.5%, respectively. Independent predictors for poorer OS were N stage, hoarseness and recurrent laryngeal nerve lymph node (RLN) involvement, and predictors for LRFFS were N stage and EQD2 (equivalent dose in 2 Gy fraction) to gross tumor volume (GTV), with ≥ 66Gy achieving local control of 94.7%. Patients receiving elective nodal irradiation (ENI) had better nodal regional control than those receiving involved field irradiation (IFI). 31 (36%) patients had treatment failure and 15 (17.4%), 8 (9.3%) and 14 (16.2%) patients had local, regional, and distant failure, respectively. 86.7% (13/15) local failures were within GTV, and supraclavicular region (62.5%, 5/8) was the most common regional failure site. No severe toxicities were observed.

**Conclusions:**

Our results seem to indicate that good locoregional control might be achieved for CEC with adequate radiation dose and treatment planning approaches.

## INTRODUCTION

Squamous cell carcinoma of the cervical esophagus (CEC) is a relatively rare entity, accounting for only 2%–10% of all esophageal cancers [1]. Because of the low incidence, the reported data of CEC are rather limited [2]. Due to its anatomical location, CEC often invades adjacent structures, including hypopharynx, thyroid gland, recurrent laryngeal nerves lymph node (RLN) and thoracic esophagus which are poor prognostic features. The optimal treatment approaches using either surgical or non-surgical approach is still controversial [3, 4]. Despite progress made using modern surgical techniques, significant postoperative complications remain, with a negative impact on the patient's quality of life. Organ-sparing definitive chemoradiotherapy (CRT) is generally recommended for CEC by current consensus guidelines [5–8].

The standard definitive radiation dose for advanced esophageal cancer is 50.4 Gy with concurrent chemotherapy [5]. However, for CEC patients, the most common failure pattern remains local-regional [9], suggesting 50.4 Gy may not be adequate. Some reports have indicated that a radiation dose of 66–70 Gy to the primary tumor, similar to the treatment of head and neck cancer, may be needed [10, 11]. Other studies have consistently reported greater local-regional control rate when higher doses were used [12, 13]. This is clinically relevant, especially with the use of modern photon-based radiotherapy techniques, such as intensity-modulated radiotherapy (IMRT) and volumetric-modulated arc therapy (VMAT). These techniques generate greater dose conformity to the target volume while allowing both the delivery of a higher dose to the tumor and better sparing adjacent organs at risk. However, the data regarding the clinical efficacy and failure pattern for CEC patients treated with modern RT techniques is still somewhat limited. Since there is still no consensus on the optimal RT dose or the extent to which prophylactic treatment of regional nodal basin needs to be included, the current study aims to investigate the outcomes, failure patterns and prognostic factors for CEC patients treated with modern definitive CRT/RT.

## RESULTS

### Patients’ characteristics

Patient characteristics and treatment factors were shown in Table 1. All CEC patients had SCC histology and the majority had stage III disease (n=71, 82.6%). The number of patients treated with simultaneous integrated boost (SIB), fraction dose >2Gy and total dose to the GTV >66 Gy were 67 (77.9%), 44 (51.2%) and 20 (23.3%), respectively. The median radiation dose for GTV was 61.6 Gy (range, 50-70 Gy) and CCRT were given to 60(69.8%) patients according to patients’ performance status and chemotherapy tolerance. Based on imaging information, 48.8% (42/86) had at baseline supraclavicular/cervical region metastasis. Among the 43 (50%, 43/86) patients with RLN metastasis, 62.8% (27/43) also had supraclavicular lymph node metastasis.

**Table 1:** Patient and treatment characteristics of CEC

Characteristics	No. of patients (%)
Age (years)	
Median	66
Range	20-87
Sex	
Male	45 (52.3%)
Female	41 (47.7%)
Smoking (pack-years)	
<20	66 (76.7%)
≥20	20 (23.3%)
Alcohol	
Not heavy drinking	5 (5.8%)
Heavy drinking	81 (94.2%)
ECOG performance status	
0-1	78 (90.7%)
2-3	8 (9.3%)
Weight loss before therapy	
<5%	54 (62.8%)
≥5%	32 (37.2%)
Weight loss during therapy	
<5%	73 (84.9%)
≥5%	13 (15.1%)
Hoarseness	
No	81 (94.2%)
Yes	5 (5.8%)
Primary tumor length	
<4cm	19 (22.1%)
≥4cm	67 (77.9%)
Hypopharyngeal extension	
No	80 (93.0%)
Yes	6 (7.0%)
RLN LN	
Negative	43 (50.0%)
Positive	43 (50.0%)
supraclavicular LN	
Negative	44 (51.2%)
Positive	42 (48.8%)
Histologic grade	
1-2	38 (44.2%)
3	48 (55.8%)
Stage	
I-II	15 (17.4%)
III	71 (82.6%)
T stage	
1-2	23 (26.7%)
3-4	63 (73.3%)
N stage	
0-1	53 (61.6%)
2-3	33 (38.4%)
Radiotherapy technique	
3D-CRT	4 (4.6%)
IMRT	52 (60.5%)
VMAT	30 (34.9%)
CTVnd delineation	
IFI	40 (46.5%)
ENI	46 (53.5%)
Dose boost schemes	
SIB	67 (77.9%)
SEQ	19 (22.1%)
Fraction dose (Gy)	
≤2	42 (48.8%)
>2	44 (51.2%)
Radiation dose for GTV (Gy)	
<66	66 (76.7%)
≥66	20 (23.3%)
Radiation dose for CTV (Gy)	
<50	13 (15.1%)
≥50	73 (84.9%)
Concurrent chemotherapy	
No	26 (30.2%)
Yes	60 (69.8%)

Abbreviations: ECOG, eastern cooperative oncology group; RLN LN, recurrent laryngeal nerve lymph node; 3D-CRT, three-dimensional conformal radiotherapy; IMRT, intensity-modulated radiotherapy; VMAT, volumetric-modulated arc therapy; GTV, gross tumor volume; CTV: clinical target volume; IFI, involved field irradiation; ENI, elective nodal irradiation; SIB, simultaneous integrated boost; SEQ, sequential boost.

### Treatment outcomes and prognostic factors

**Figure 1:**
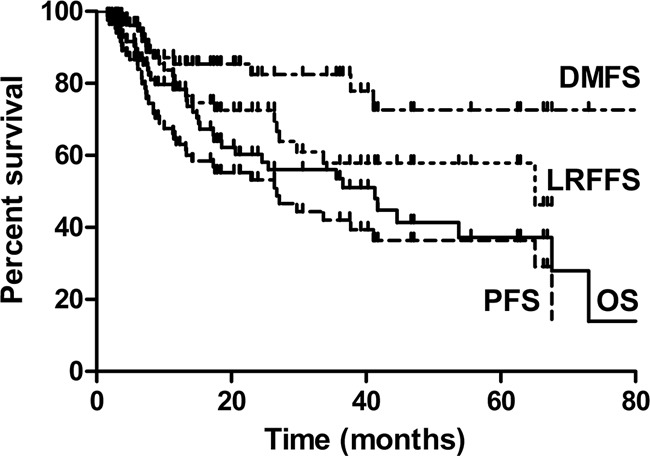
Kaplan Meier analysis for Overall survival (OS), local-regional failure-free survival (LRFFS), distance metastasis free survival (DMFS) and progression free survival (PFS) of cervical esophageal cancer (CEC)

**Figure 2:**
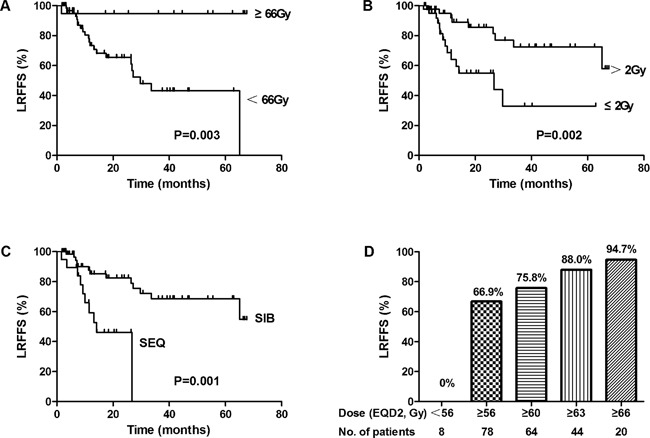
Relationship of LRFFS and radiotherapy (RT) schemes (A) Radiation dose for GTV **(B)** Fraction dose. **(C)** Dose boost method (SIB, simultaneous integrated boost; SEQ, sequential boost). **(D)** Relationship of radiation dose for GTV and LRFFS.

The median follow-up time was 19.4 months (range, 1.6-80.1 months). The 3-year OS, LRFFS, DMFS and PFS rate for all patients were 53.6%, 57.9%, 81.5%, and 41.5%, respectively (Figure 1). The median OS, LRFFS and PFS were 41.3 months, 65.1 months and26.7 months, respectively. Univariate analysis for clinical factors that influenced outcomes was shown in Supplementary Table 1. Patients with ≥5% weight loss before treatment and hoarseness were significantly associated with worse OS and PFS, and higher level of weight loss during therapy (≥5%) was significantly associated with worse DMFS. Positive RLN seen on diagnostic imaging were significantly associated with worse OS, DMFS and PFS, whereas lower N stage (0-1) was associated with significantly better OS, LRFFS, DMFS and PFS. Furthermore, higher total EQD2 dose to the GTV was associated with better LRFFS and PFS, higher fraction dose (>2 Gy) with better OS, LRFFS, DMFS and PFS, and SIB with better DMFS (Also seen in Figure 2A-2C). We further analyzed the correlation between LRFFS and total EQD2 dose to GTV. As shown in Figure 2D, there was a trend towards an increase in LRFFS when the EQD2 dose increased from 56Gy to 66Gy, with >66Gy achieving a LRFFS rate of 94.7%.

In multivariate analysis (Table 2), N stage was independent prognostic indicator for OS (P=0.001, HR=3.258), LRFFS (P=0.001, HR=5.219) and PFS (P<0.001, HR=3.854), radiation dose for GTV (cutoff of 66Gy) was independent prognostic indicators for LRFFS (P=0.009, HR=0.064) and PFS (P=0.018, HR=0.330), hoarseness and RLN status were independent prognostic indicators for OS (P=0.037, HR=2.817) and DMFS (P=0.048, HR=3.272), respectively.

**Table 2:** multivariate analysis of prognostic factors on treatment results for CEC

Endpoint	Prognostic factors	Multivariate analysis	
		*P*	HR (95%CI)
3y OS	Hoarseness (Negative *vs* Positive)	0.037	2.817 (1.062-7.472)
	N stage (0-1 *vs* 2-3)	0.001	3.258 (1.648-6.444)
3y LRFFS	N stage (0-1 *vs* 2-3)	0.001	5.219 (2.010-13.548)
	GTV dose (<66Gy *vs* ≥66Gy)	0.009	0.064 (0.008-0.502)
3y DMFS	RLN LN (Negative *vs* Positive)	0.048	3.272 (1.011-10.587)
3y PFS	Hoarseness (No *vs* Yes)	0.100	2.256 (0.856-5.945)
	N stage (0-1 *vs* 2-3)	<0.001	3.854 (2.000-7.428)

Abbreviations: OS, overall survival; LRFFS, local-regional failure-free survival; DMFS, distance metastasis free survival; PFS, progression-free survival; HR, hazard ratio; 95% CI, 95% confidence interval.

### Failure patterns

At the last follow-up visit, a total of 31 patients had treatment failure and 21 patients (24.4%) experienced local-regional failure, among which15 (17.4%), 8 (9.3%) and 14 (16.2%) patients had developed local failure, regional failure, and distant metastasis, respectively (Table 3). The median time to local failure, regional failure and distant metastasis was 9.3 (2.5-65.1), 11.8 (3.5-27.1) and 6.7 (1.6-41.1) months, respectively. A total of 38 patients died at the end of the study. Causes of death included local failure, regional failure, distant metastasis and other non-tumor factors.

**Table 3:** Incidence and site of failure

	N (%)
Local only	12 (38.7%)
Local and regional	2 (6.4)
Local and distant	1 (3.2%)
Local, regional and distant	0
Regional only	3 (9.7%)
Regional and distant	3 (9.7%)
Distant only	10 (32.3%)

**Figure 3:**
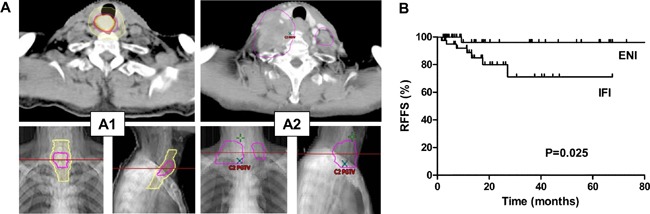
Local-regional nodal failure after radiotherapy and the omission of prophylactic nodal coverage **(A)** Example of supraclavicular LN metastasis cases after treatment. A1: Primary tumor (highlight orange) identified by positron emission tomography-computed tomography (PET-CT), including both gross tumor volume (GTV, magenta) and clinical target volume (CTV, yellow); A2: The occurrence of supraclavicular LN metastasis after treatment (fuchsia). **(B)** Relationship of regional failure-free survival (RFFS) and CTVn delineation method. ENI, elective nodal irradiation, IFI, involved field irradiation.

Among 15 patients with local failures, most local failure sites were located in GTV (86.7%, 13/15) and others were located in CTV (13.3%, 2/15). Eight patients had regional failures, among whom 2 had failures within the CTV and 6 out of CTV. Supraclavicular area (62.5%, 5/8) was the most common regional failure site and all of them failed outside of the CTV (Figure 3A). None of patients recurred in RLN chains region. In addition, the relationship between regional failure-free survival (RFFS) and nodal clinical target volume (CTVnd) delineation was analyzed; Patients who received elective nodal irradiation (ENI) had better RFFS than those who received involved field irradiation (IFI) (Figure 3B).

The most common metastatic sites were lung (9/14, 64.3%), liver (4/14, 28.6%), bone (2/14, 14.3%), axillary or lower abdomen lymph nodes (2/14, 14.3%) and brain (1/14, 7.1%). Three patients had multiple metastases.

### Toxicities

The most commonly observed acute toxicities, which were mainly grades 1 or 2, included laryngopharyngeal and esophageal mucositis, leucopenia, thrombocytopenia, skin and gastrointestinal reactions. The incidence of acute grade 3 mucositis, leucopenia and thrombocytopenia were 2.3% (2/86), 16.3% (14/86) and 3.5% (3/86), respectively. No patient developed acute grade 4 toxicity, and there were no treatment-related deaths.

## DISCUSSION

Due to the low incidence of CEC, reported data on the definitive radiotherapy management of CEC is rather limited. In some older series, the typical 3-year OS rate was less than 35% [17]. In more modern series, the 3-year OS rates appear to have been improved to nearly 40% [18, 19]. In the current study, the reported 3-year OS was 53.6%, suggesting that in the modern era, the employment of multiple multidisciplinary advances, including possibly advances in RT techniques, may play roles in improving outcomes of CEC [20, 21].

Local-regional failure rates are high for CEC after definitive radiotherapy or chemoradiotherapy, with the local control rate of approximately 58-68% [14, 18]. For our study, the 3-year LRFFS was 58% and 24% of the patients experienced loco-regional failure at the last follow up, which was the major failure pattern. The need to escalate the radiation dose in order to improve the local-regional control remains unclear. The only randomized controlled trial that tried to answer the need for dose escalation by comparing the standard 50.4 Gy to the higher 64.8 Gy [22] indicated no clinical benefit for dose escalation among EC patients treated with CCRT. However, since more than 85% patients had adenocarcinoma in various tumor location sites, it may be inappropriate to extrapolate the results of the study to CEC with SCC using modern RT technologies. Some recent retrospective studies suggested that higher dose could lead to better LRFFS and even OS in CEC patients [9, 19], and another study also suggested that for the squamous CEC, dose greater than 50.4 Gy may help improve tumor local control [23]. One study has shown that dose escalation in esophageal cancer based on 18FDG-PET/CT could be achieved up to70Gy safely by SIB technique [11]. This is especially the case in our study and we found EGD2 dose for GTV (cutoff value 66Gy) was independent prognostic indicators for LRFFS. There was a trend towards an increase in LRFFS when the EQD2 dose was increased from 56Gy to 66Gy, with 66Gy and above achieving a high LRFFS rate of 94.7%, which was in accordance with one study showing that the 2-year OS rate was significantly better in the patients receiving 66 Gy (55.6% vs 37.5%) [19]. In this study, 77.9%, 51.2% and 23.3% patients were treated with SIB, fractionation dose >2Gy and total dose for GTV >66 Gy, respectively, and might partially account for the relatively higher survival outcomes in this study. Concurrent chemoradiotherapy is the standard of care for locally advanced unresectable or inoperable esophageal cancer patients based on intergroup studies [22, 24]. Although 30.2% of patients in the current study didn't get concurrent chemotherapy due to poor performance status or intolerance, it did not translate to worse outcome by univariate analysis. One explanation might be that the median dose for patients not receiving chemotherapy was as high as 64Gy (range 50.4-70Gy), which may have compensated for the omission of concurrent chemotherapy. Taken together, the data seems to indicate that the treatment outcomes of CEC may improve with higher doses, with our data indicating doses to the GTV>66Gy with fractionation dose >2Gy by SIB technique may improve local control, much like for the management for head and neck cancers. Certainly randomized trials comparing high dose vs. low dose will be necessary to confirm our observation.

Regional lymph node metastasis was shown to be a predictive factor for outcome of EC in the setting of surgery [25, 26]. Many studies concerning radical esophagectomy showed that the number of metastatic LNs was independent predictive factor for EC prognosis [27], which lays the basis for the N stage in the 7th edition AJCC TNM classification. Although the N-stage for the current study was done clinically and not surgically, we still found that N stage was an independent prognostic factor for OS, LRFFS and PFS, which was not predictive in some studies using 6th edition AJCC classification [14, 17–19].

Currently, there is no consensus regarding the delineation of CTV for CEC, which in principle is the coverage of potentially microscopic areas of spread of disease. For upper EC patients, RLN chains and supraclavicular/cervical region were the most common LN metastasis regions after esophagectomy, and the reported LN metastatic rates were 43.3% and 46.2% respectively [28]. In the setting of esophagectomy, three field lymph node dissection was strongly recommended for upper EC including CEC [28], even for middle or lower thoracic EC with positive RLN, bilateral supraclavicular LN dissection or RT was considered in order to enhance the regional control rate [29, 30]. Based on imaging information, similar result was found in this study, with 48.8% (42/86) of patients with supraclavicular/cervical region metastasis, and among the 50% (43/86) of patients with RLN metastasis, 62.8% (27/43) also had supraclavicular lymph node metastasis. RLN metastasis was independent prognostic indicators for OS and DMFS in our study. We also found that regional failure sites were mainly located in the supraclavicular area (62.5%, 5/8) and all of them failed outside of CTV if they were not prophylactically covered (or so-called “electively treated”). Moreover, our study showed that, hoarseness, which is the main clinical manifestations caused by RLN tumor invasion or RLN compression, was an independent prognostic factor for OS, which was also reported in another study [18]. Therefore, the RLN chains and supraclavicular/cervical region treatment should be prophylactically covered if not already involved, as evidenced by the fact that patients receiving ENI had significantly better RFFS than those receiving IFI. Although the need for elective coverage of the supraclavicular and upper mediastinal regions is still somewhat controversial [31, 32], the nodal metastatic patterns after surgery and RT [5, 33, 34], as well as data from the current study, suggest prophylactic irradiation of the supraclavicular and upper mediastinal RLN chains regions should be recommended.

Due to its retrospective nature, this study has some limitations including the potential confounding factors and small patient numbers which might affect the final conclusion of the study. Furthermore, the single institutional nature of the study may also limit the applicability of our findings. Therefore, well-designed, larger multi-center prospective trials may be needed.

In conclusion, our experience suggested that definitive RT using modern techniques was effective for CEC with low toxicities, but local-regional failure was still the most common failure pattern. N stage, radiation dose for GTV, hoarseness and RLN status were independent prediction factors for outcomes. Higher GTV dose of 66Gy or above and prophylactic irradiation of supraclavicular and upper mediastinal RLN regions may help improve local-regional control.

## MATERIALS AND METHODS

### Patients

Between November 2007 and May 2015, 86 consecutive CEC patients received definitive CRT/RT at the Department of Radiation Oncology in Xijing Hospital, Fourth Military Medical University. We defined the region of the primary tumor to lie between the cricopharyngeus muscle and the thoracic esophagus inlet [14]. At the time of diagnosis, patients had histologically proven squamous cell EC, Karnofsky performance score ≥ 70, and no evidence of distant metastasis. CEC patients were staged according to the 7th edition American Joint Committee on Cancer (AJCC) staging classification. Patient characteristics and treatment factors are shown in Table 1. This study was approved by the ethics committee of Xijing Hospital.

### Treatment

Patients were treated with either three-dimensional conformal radiotherapy (3D-CRT) (n=4), IMRT (n=52) or VMAT (n=30). For radiotherapy, gross tumor volume (GTV) was defined as the primary tumor (GTVt) and involved lymph nodes (GTVnd) based on all available information deriving from barium swallow, laryngoscopy, contrast enhanced neck/chest computed tomography scan (CT), endoscopy/EUS and 18FDG-positron emission tomography (PET)-CT. The prescription dose to GTV ranged from 50 to 70 Gy in 25 to 35 fractions with five fractions per week over 5-7 weeks, covering both GTVt and GTVnd. Sequential or simultaneous integrated boost (SIB) approach was applied for GTVt and GTVnd dose escalation. The clinical tumor volume (CTV) comprised the GTVt plus additional 3cm cranial-caudal and 0.7-1cm radial margin expansions, respectively, as well as involved or elective nodal regions, which included supraclavicular fossa and upper mediastinal areas, with the prescription dose of 50-54 Gy. In order to account for daily set-up errors during treatment, the planning target volume (PTV) was created with a 0.5-1.0 cm margin from GTV and CTV respectively, which were named as PGTV (PGTVt and PGTVnd) and PCTV (PCTVt and PCTVnd), respectively. Detailed definition of Involved Field Irradiation (IFI) and definition of Elective Nodal Irradiation (ENI) were shown in (Supplementary Figure 1A and 1B).

Most patients received concurrent chemoradiation therapy (CCRT). Patients were treated with radiotherapy alone due to poor KPS or intolerance to chemotherapy. The chemotherapy regimens mainly included combination of cisplatin and 5-fluorouracil (47/60, 78.3%), oral capecitabine or S1 alone (13/60, 21.7%).

### Follow-up

All patients were monitored weekly during treatment and evaluated every 3 months in the first 2 years and every 6 months thereafter. Each follow-up included a physical examination, routine blood count and chemistries, barium swallow, ultrasonography of neck and abdomen, and contrast enhanced CT scans of the neck and thorax. Endoscopy with or without biopsy was recommended every 6 months or as clinically indicated. Radiotherapy-related toxicities were evaluated and scored on a weekly basis according to the acute and the late-onset radiation morbidity scoring criteria of the Radiation Therapy Oncology Group. 3 months after the treatment, late toxicities were evaluated.

### Statistical analysis

All radiation doses were converted into the equivalent dose in 2 Gy fraction (EQD2), using an α/β=10 and calculated using the prescribed dose×(10+ dose per fraction)/12. Treatment failure was either confirmed pathologically or documented radiographically by serial progression [15]. Local and regional failure was defined as the persistence or recurrence of the primary tumor or regional lymph nodes, respectively [16]. Distant metastatic failure was defined as metastasis to any site beyond the primary tumor and regional lymph nodes. Progression-free survival (PFS), loco-regional failure-free survival (LRFFS), distance metastasis free survival (DMFS), overall survival (OS) were defined as the time from the first date of treatment until the date of tumor progression, local-regional tumor persistence or recurrence, distant recurrence and death, respectively. OS, LRFFS, DMFS, and PFS were calculated by Kaplan-Meier method. The log-rank tests were also used for univariate analyses to select potential prognostic factors. We used p value less than 0.10 as cutoff to screen the factors for the subsequent multivariate analyses. Cox proportional hazard model was used for multivariate analysis, using significance cutoff of α=0.05. All statistical analyses were performed by SPSS 18.0 (SPSS, Chicago, IL, USA).

## SUPPLEMENTARY MATERIALS FIGURE AND TABLE




