# Supplementary material for: Patterns of failure and clinical outcomes of definitive radiotherapy for cervical esophageal cancer
Source: Oncotarget. 2017 Feb 24;8(13):21852–60. doi: 10.18632/oncotarget.15665 (PMC5400628; doi:10.18632/oncotarget.15665)
Supplement: Supplementary file 1 [file oncotarget-08-21852-s001.pdf]

Patterns of failure and clinical outcomes of definitive radiotherapy for cervical esophageal cancer

SUPPLEMENTARY MATERIALS

SUPPLEMENTARY FIGURE AND TABLE

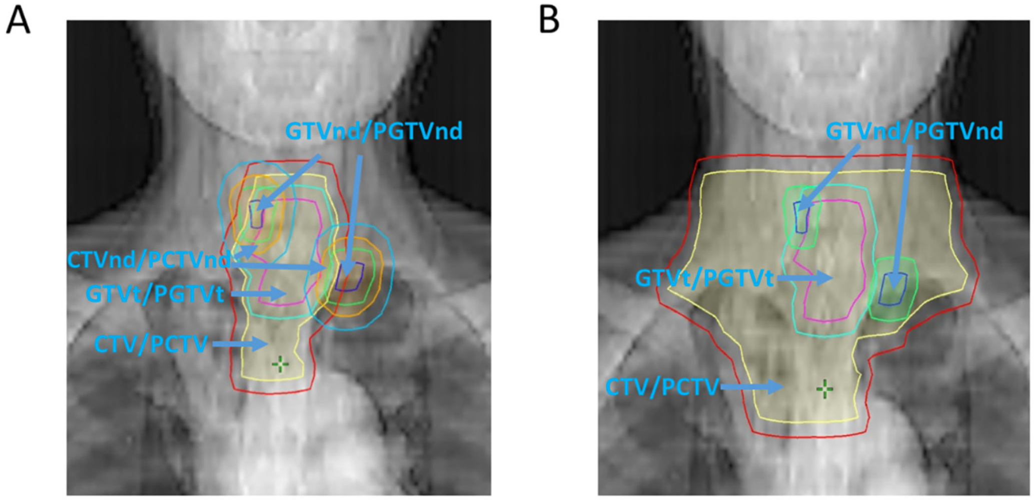

Supplementary Figure 1: Schematic diagram of CTV delineation and field design. IFI **(A)** and ENI **(B)** field in the coronal direction. **Definition of Involved Field Irradiation (IFI):** The gross tumor volume (GTV) included GTVt (tumor) and GTVnd (lymph node), which was defined as any visible tumors or positive lymph nodes based on upper endoscopy and endoscopic ultrasound evaluations or were shown on barium esophagogram, CT scan with contrast, positron emission tomography (PET). The clinical target volume (CTV) consisted of CTVt and CTVnd. CTVt was defined as the GTVt plus additional 3cm cranial-caudal and 0.7-1cm radial margins, and CTVnd was defined as GTVnd plus 0.5-1.0 cm radial margin. In order to account for daily set-up errors during treatment, the planning target volume (PTV) was created with a 0.5-1.0 cm margin from GTV and CTV respectively, which were named as PGTV (PGTVt and PGTVnd) and PCTV (PCTVt and PCTVnd), respectively. **Definition of Elective Nodal Irradiation (ENI):** The definition for GTVt, GTVnd and CTVt were the same as that of IFI. CTV included CTVt, positive lymph node region and the areas at risk as elective nodal regions, including supraclavicular, upper mediastinal regions adjacent to the primary tumors. The PTV definition was the same as that of IFI.

Supplementary Table 1: Univariate analysis of prognostic factors on treatment results for CEC.

See Supplementary File 1
