# Supplementary material for: Patterns of failure and clinical outcomes of definitive radiotherapy for cervical esophageal cancer
Source: Oncotarget. 2017 Feb 24;8(13):21852–60. doi: 10.18632/oncotarget.15665 (PMC5400628; doi:10.18632/oncotarget.15665)
Supplement: Supplementary file 2 [file oncotarget-08-21852-s002.docx]

Table 1. Univariate analysis of prognostic factors on treatment results for CEC

| Characteristics | 3-year OS | 3-year LRFFS | 3-year DMFS | 3-year PFS |
| --- | --- | --- | --- | --- |
| Age (years) | *p* = 0.205 | *p* = 0.930 | *p* = 0.833 | *p* = 0.781 |
| ＜66 | 61.3% | 55.1% | 82.2% | 39.5% |
| ≥66 | 47.1% | 60.7% | 81.1% | 43.5% |
| Sex | *p* = 0.945 | *p* = 0.255 | *p* = 0.472 | *p* = 0.578 |
| Male | 53.5% | 49.7% | 77.7% | 36.0% |
| Female | 53.7% | 69.4% | 86.6% | 48.1% |
| Smoking (pack-years) | *p* = 0.109 | *p* = 0.909 | *p* = 0.236 | *p* = 0.133 |
| ＜20 | 46.2% | 56.4% | 76.8% | 35.5% |
| ≥20 | 53.3% | 62.1% | 78.9% | 40.7% |
| Alcohol | *p* = 0.138 | *p* = 0.102 | *p* = 0.728 | *p* = 0.185 |
| rarely | 50.0% | 53.3% | 79.9% | 36.8% |
| often | 55.5% | 62.4% | 75.0% | 42.3% |
| ECOG performance status | *p* = 0.733 | *p* = 0.575 | *p* = 0.299 | *p* = 0.854 |
| 0-1 | 54.9% | 58.0% | 75.6% | 41.6% |
| 2-3 | 35.7% | 50.0% | 70.0% | 35.7% |
| Weight loss before therapy | *p* = 0.034 | *p* = 0.091 | *p* = 0.693 | *p* = 0.037 |
| ＜5% | 65.2% | 64.1% | 81.5% | 49.4% |
| ≥5% | 31.3% | 43.1% | 81.7% | 25.2% |
| Weight loss during therapy | *p* = 0.577 | *p* = 0.021 | *p* = 0.596 | *p* = 0.368 |
| ＜5% | 57.7% | 62.2% | 80.6% | 44.2% |
| ≥5% | 19.0% | 35.4% | 80.7% | 26.5% |
| Hoarseness | *p* = 0.005 | *p* = 0.270 | *p* = 0.083 | *p* = 0.010 |
| No | 58.7% | 59.7% | 83.7% | 44.8% |
| Yes | 0% | 33.3% | 50.0% | 0% |
| Primary tumor length | *p* = 0.391 | *p* = 0.924 | *p* = 0.647 | *p* = 0.568 |
| ＜4cm | 57.0% | 50.8% | 82.1% | 38.4% |
| ≥4cm | 53.7% | 61.2% | 81.7% | 44.1% |
| Hypopharyngeal extension | *p* = 0.147 | *p* = 0.897 | *p* = 0.150 | *p* = 0.275 |
| No | 56.2% | 57.6% | 83.5% | 42.9% |
| Yes | 20.8% | 66.7% | 55.6% | 22.2% |
| RLN LN status | *p* = 0.019 | *p* = 0.192 | *p* = 0.037 | *p* = 0.008 |
| Negative | 63.8% | 65.2% | 88.6% | 27.4% |
| Positive | 42.5% | 49.0% | 73.0% | 55.1% |
| Histologic grade | *p* = 0.240 | *p* = 0.197 | *p* = 0.100 | *p* = 0.160 |
| 1-2 | 72.7% | 64.2% | 85.7% | 54.1% |
| 3 | 56.5% | 64.3% | 81.3% | 48.7% |
| AJCC Stage | *p* = 0.435 | *p* = 0.403 | *p* = 0.221 | *p* = 0.148 |
| Ⅰ-Ⅱ | 52.1% | 64.0% | 90.0% | 54.8% |
| Ⅲ | 50.8% | 56.4% | 79.3% | 38.8% |
| T stage | *p* = 0.387 | *p* = 0.317 | *p* = 0.583 | *p* = 0.664 |
| 1-2 | 55.6% | 48.6% | 88.0% | 38.6% |
| 3-4 | 39.8% | 61.2% | 79.1% | 42.6% |
| N stage | *p* ＜0.001 | *p* = 0.001 | *p* = 0.019 | *p* ＜0.001 |
| 0-1 | 68.5% | 69.6% | 88.8% | 56.0% |
| 2-3 | 26.4% | 30.3% | 63.2% | 15.8% |
| Radiotherapy technique | *p* = 0.413 | *p* = 0.061 | *p* = 0.588 | *p* = 0.355 |
| 3D-CRT | 75.0% | 25.0% | 50.0% | 25.0% |
| IMRT | 49.9% | 65.8% | 77.4% | 41.5% |
| VMAT | 60.5% | 59.3% | 85.4% | 56.7% |
| CTVn delineation | *p* = 0.227 | *p* = 0.524 | *p* = 0.459 | *p* = 0.404 |
| IFI | 46.3% | 53.0% | 76.6% | 36.2% |
| ENI | 61.0% | 61.2% | 86.1% | 46.1% |
| Dose boost schemes | *p* = 0.351 | *p* = 0.001 | *p* = 0.983 | *p* = 0.119 |
| SIB | 56.9% | 68.5% | 82.0% | 47.4% |
| SEQ | 34.5% | 0.00% | 81.7% | 0.00% |
| Fraction dose (Gy) | *p* = 0.027 | *p* = 0.002 | *p* =0.041 | *p* = 0.026 |
| ≤2 | 36.9% | 33.0% | 65.2% | 22.4% |
| ＞2 | 63.9% | 72.5% | 92.3% | 53.3% |
| GTV dose (Gy) | *p* = 0.097 | *p* = 0.003 | *p* = 0.859 | *p* = 0.022 |
| ＜66 | 46.1% | 43.1% | 82.2% | 31.7% |
| ≥66 | 79.5% | 94.7% | 83.1% | 72.1% |
| CTV dose (Gy) | *p* = 0.704 | *p* = 0.126 | *p* = 0.874 | *p* = 0.102 |
| ＜50 | 46.2% | 0.00% | 80.9% | 0.00% |
| ≥50 | 54.4% | 63.2% | 81.4% | 46.4% |
| Concurrent chemotherapy | *p* = 0.916 | *p* = 0.548 | *p* = 0.629 | *p* = 0.815 |
| No | 49.7% | 51.4% | 77.6% | 31.9% |
| Yes | 56.4% | 62.0% | 83.9% | 47.6% |

Abbreviations: ECOG, eastern cooperative oncology group; RLN LN, recurrent laryngeal nerve lymph node; 3D-CRT, three-dimensional conformal radiotherapy; IMRT, intensity-modulated radiotherapy; VMAT, volumetric-modulated arc therapy; GTV, gross tumor volume; CTV: clinical target volume; CTVn, nodal clinical target volume; IFI, involved field irradiation; ENI, elective nodal irradiation; SIB, simultaneous integrated boost; SEQ, sequential boost.
